# Seizing the opportunity: the emergence of shared leadership during the deployment of an integrated performance management system

**DOI:** 10.1186/s12913-022-07690-3

**Published:** 2022-03-02

**Authors:** Pierre-Luc Fournier, Line Moisan, Denis Lagacé

**Affiliations:** 1 Department of Information Systems and Quantitative Methods, Business School, Université de Sherbrooke, 2500 boul. de l’Université, Sherbrooke, J1K 2X9 Canada; 2 Chaire Interdisciplinaire de Recherche et d’Intervention dans les Services de Santé, Université du Québec à Trois-Rivières, 3351 boul. des Forges, Trois-Rivières, G8Z 4M3 Canada; 3 Department of Industrial Engineering, School of Engineering, Université du Québec à Trois-Rivières, 3351 boul. des Forges, Trois-Rivières, G8Z 4M3 Canada

**Keywords:** Leadership, Distributed leadership, Performance management system, Collaboration, Control rooms, Status sheets

## Abstract

**Background:**

Performance management systems have been introduced in health and social services institutions to improve organizational performance, supporting the emergence of new management behaviors that are more rooted in collaborative management practices. This study aims to understand how different leadership styles emerge through the implementation of a performance management system and its related tools, and how these can foster distributed leadership.

**Methods:**

Over two years, the implementation of an integrated performance management system supporting the integration of social services for children, youth, and families was studied at a recently merged Canadian healthcare organization. Qualitative analysis of data collected from 15 interviews, 3 focus groups, and over 350 h of non-participant observation was conducted.

**Results:**

The results show that leadership evolved to adapt to the context of organizational integration and was no longer confined to a single manager. Transformational leadership was needed to encourage the emergence of a new integrated performance management system and new behaviors among middle managers and team members. Transactional leadership was legitimized through the use of a status sheet when the integration project did not deliver the expected results. Both transformational and transactional leadership paved the way to distributed leadership, which in turn promoted collaborative practices associated with activities in control rooms and dialogue stemming from the status sheets. Distributed leadership among team members made a difference in the outcome of the integration project, which became a driver of collaboration.

**Conclusions:**

The integrated performance management system and the use of its tools can help renew leadership in health and social service organizations. The results lend credence to the importance of distributed leadership in promoting collaborative practices to improve services for children, youth, and families. The results also highlight how various leadership styles can contribute to the emergence of distributed leadership over time.

## Introduction

In recent decades, health and social service organizations have attempted to implement various initiatives to improve performance [1], particularly by engaging in integration mechanisms [2] involving unpredictable changes that can disturb an organization [3]. Integrating health and social services is a major challenge [4], and the World Health Organization [5] sees this integration as a way to improve the performance of services in terms of access, quality, and client satisfaction while emphasizing the importance of interprofessional collaboration [6–8]. To achieve this goal, health and social service institutions have introduced performance management systems to support their organizational performance [9]. An integrated performance management system is one of the foundations for integrating care and services through multidisciplinary teamwork [10, 11].

A condition for successful service integration is collaboration, which is often presented as a tool to find new solutions to the complex problems of the fragmentation of care and services [12] and to resolve the porosity of organizational boundaries and structures. In light of this trend, the role of the leader is evolving from a process of vertical influence to one of distributed leadership among team members [13, 14].

On April 1, 2015, the province of Quebec, Canada, restructured its health and social services network by reducing its number of institutions from 182 to 34. These new institutions now group the facilities and services for the population of its region. The initial goal of the reform was to reduce bureaucracy by flattening organizational structures from three to two levels and centralizing powers with the Minister of health and social services. This flattening of the management hierarchy resulted in the elimination of 1300 managerial positions.^1^ To pursue the performance improvement of its network, the Quebec health and social services ministry [15] included a requirement in its strategic planning^2^ that 100% of its institutions would have to deploy a strategic control room by the end of 2018 [16]. The Ministry also expected tactical and operational rooms to be implemented by March 30, 2020. Control rooms are tools of an integrated performance management system (IPMS) and, according to Moisan et al. [11], the adoption of this system represents a true mechanism for value creation.

The transformation of the health and social services network is supported by the deployment of a new performance management system that encourages stakeholders to reflect on the evolving role of managers. An IPMS encourages traditional models, in which leadership resides in a single individual, to evolve into leadership development efforts that extend to all levels of the organization. Transformational leadership and transactional leadership are powerful concepts for improving organizational performance [17, 18]. However, distributed leadership is potentially a more useful solution for team management compared to hierarchical and vertical leadership [19, 20].

This paper studies how different leadership styles evolve during the deployment of an IPMS. Through a longitudinal case study, it aims to demonstrate how the adaptation of different leadership styles in conjunction with IPMS tools can support the integration of social services for children, youth, and families. The following section draws from a literature review to summarize the main leadership typologies and presents the integrated performance management system and the various tools used by the organization. This is followed by a presentation of the organizational context and the research methodology. The results reveal a multi-dimensional experience of how leadership styles evolved over more than two years. The paper concludes with a discussion of the results, the implications, and future avenues of research.

### Background – different leadership styles

Over the last forty years, the literature on leadership has evolved from the “Great Person Theory” into how leaders behave and develop relationships with followers [21]. According to Northouse [22], there are two categories of leader behaviors: task behaviors are focused on “getting the work done” and ensuring objectives are met, while relationship behaviors are focused on helping the group function properly. Burns [23] was the first to put forward the concepts associated with transformational and transactional leadership. Bass [24] builds on this work and focuses primarily on transformational leadership, which leads to the development of more engaged and dedicated employees who can achieve a high level of performance [24]. The works of Burns [23] and Bass [24] present a typology of three main leadership styles—laissez-faire, transactional and transformational leadership—, each of which has its distinct characteristics.

#### Laissez-faire leadership

Laissez-faire leadership is a passive form of leadership, where leaders provide little to no feedback which can often leave employees in the dark. This is usually detrimental to employee engagement. This type of leader is nowhere to be seen when problems arise and is not very engaged with his or her employees. These leaders seek a hands-off approach to management [25].

#### Transactional leadership

Transactional leaders focus on the resources they manage. They also employ management by exception either in an active way (trying to prevent problems) or a passive way (only intervening when a problem occurs) [26]. Transactional leaders are usually focused on getting results [22], and typically work under the assumption that they know how employees should behave for the organization to reach its goals [27].

#### Transformational leadership

Transformational leaders are portrayed as charismatic and motivating managers who challenge *(intellectual stimulation)* and care (*individual consideration*) about each employee, who can articulate a vision that inspires employees, and foster the acceptance of group goals [28, 29]. Transformational leaders can also connect observable behaviors with this vision to reinforce organizational change capacity [30]. Transformational leader behaviors such as *individual consideration* and *intellectual stimulation* focus on individuals, while behaviors such as *articulating a vision* and *fostering acceptance for group goals* focus on teams [31, 32].

Transformational leadership has been linked to various positive outcomes in organizations. It has been shown to positively impact how meaningful employees find their work [33, 34], which results in them being more engaged in their work [34], while also improving job satisfaction, commitment to the organization [35, 36], and employee well-being [37]. It has also been linked with voicing and organizational citizenship behaviors that are beneficial to the organization [31, 33, 38]. Transformational behaviors also favor the development of close and deep relationships between leaders and followers [39], as well as increased internal and external social capital in leadership teams [40], which can all lead to positive performance outcomes [39, 40]. This type of leadership has shown to be effective when individuals and groups identify with their leader [41, 42], notably leading to higher levels of individual and team creativity [31, 32]. However, transformational leadership tends not to be as effective when employees endorse traditional hierarchical roles and relationships [38], in which case their behaviors are likely to be a function of their role as opposed to leader behaviors. Perceived power and direct hierarchy can also minimize the effect of transformational leadership on followers [43]. Furthermore, transformational leaders tend to be more effective in environments where face-to-face dialog is possible [44].

The literature shows that transformational leadership enhances or strengthens transactional leadership [45] and that transactional and transformational behaviors are necessary for leader effectiveness [46].

#### Distributed leadership

The scope of the leadership literature, however, is not limited to transactional and transformational behaviors. While it does not replace traditional leadership behaviors, distributed leadership acknowledges the various sources of leadership through an integrated perspective [47]. Distributed leadership is an emergent phenomenon, in which leadership goes beyond the individual and is viewed as a collective property of organizations [48].

A comprehensive literature review shows a multitude of definitions of distributed leadership that add to some confusion around the concept [49, 50]. However, three main aspects can be rooted out: 1) distributed leadership supports a horizontal dynamic among team members; 2) distributed leadership is an emergent team phenomenon; 3) that the different roles of leadership are distributed among all members of a team [51]. Distributed leadership is described as a dynamic and interactive influence process among individuals in groups whose goal is to help each other reach group or organizational goals or both [52, 53].

Teams with distributed leadership should benefit in many ways from better coordination and collaboration through increased engagement of team members [54] and, incidentally, increased employee life satisfaction [55]. Teams with distributed leadership typically have less conflict, more consensus, and higher intragroup trust and cohesion [56]. Communication [57] and organizational culture [58] are considered essential for leadership to emerge within groups. Hence, the work environment and its organizational context play a key role in the emergence of leaders [58, 59].

Overall, distributed leadership is a relational and collaborative leadership process involving teams or groups that mutually influence each other and collectively share tasks and responsibilities otherwise delegated to a single leader with centralized power [60]. This model, therefore, supports effective collaborative work between different professionals working toward a common goal. Distributed leadership is also thought to have a positive impact on public sector performance [61].

#### The integrated performance management system (IPMS)

A performance management system is a process that aligns an organization strategically, tactically, and operationally and allows it to assess the effectiveness and efficiency of its processes by facilitating information flow [62, 63]. In the health and social services sector, a management system improves care and services and increases accessibility while slowing cost increases [64]. The IPMS supports a set of leadership skills, practices, and behaviors whose goal is to empower employees by encouraging their autonomy, teamwork, and problem-solving abilities [65–67].

A performance management system is therefore considered a core “habit” in high-value healthcare organizations [68]. This system also supports the concept of the quadruple aim of performance as put forward by Bodenheimer and Sinsky [69]. The quadruple aim includes enhancing the patient experience, improving population health of a given territory, reducing costs, and improving work-life for all staff. These are essential pathways to improve the performance of the health and social services system. The integrated performance management system has many tools to support continuous improvement initiatives. Of particular interest for this research are control rooms and the status sheet, both of which are presented next.

#### Control rooms and status sheet

Control rooms, also known as *obeyas*, are spaces where members of a team convene to evaluate and discuss performance gaps while assessing ways to improve [70]. Control rooms originate from Lean Thinking and were initially employed at Toyota [71]. They are typically based in large meeting rooms where visual management and problem-solving tools such as A3 reports are employed to review and improve performance during regularly occurring meetings. They can take place at all levels of the organization (strategic, tactical, and operational). In healthcare, control rooms were first adapted and experimented with at the ThedaCare Healthcare Network in the USA [72]. Based on high levels of collaboration between team members [73], control rooms foster cross-functional management to allow an organization to tackle various organizational challenges [74].

The status sheet [75] promotes dialogue to help staff learn about and better understand the organization’s operations. It guides a dialogue comprising about ten standard questions and lasting up to 20 min between an employee and his or her immediate superior, according to a standard schedule. The specific questions in the status sheet aim to support proactive management and move from superficial conversations to essential information that can prevent and avoid potential problems. As a tool, the status sheet differs from the control room in that it encourages individual discussion, whereas the control room relies on the strength of teamwork to achieve targets.

#### Context

The institution studied in this research was created in April 2015 following the merger of seven distinct organizations, which created new work environments that have become increasingly complex to manage. It oversees a vast territorial service network of 49 facilities spread out over more than 20,000 km^2^. It has 118 managers (top and middle), 3500 employees, and 220 doctors, with an annual budget of $300 million.

A longitudinal case study was conducted within the child and youth programs of this organization, which have a combined staff of over 125 multidisciplinary professionals. During the 2015 merger, 50% of middle management positions in this sector were cut, which led to a period of instability, a lack of coordination between different services, insecurity among employees that increased sick leave costs, increased bureaucracy, and decreased organizational flexibility [76]. This significant reduction required the organization to reflect on how to renew the leadership of top (directors) and middle management. The organization could no longer consider managers as the only leaders of a team with all the answers. However, nurturing bottom-up leadership was also no longer an option, as this approach was limited and not suited to the organizational context [76, 77].

This qualitative study took place over two years and aimed to answer the following question: “How do different leadership styles evolve with the implementation of an integrated performance management system and its tools?”

## Methods

A single longitudinal case study was used in this research. The case study is a collection of observations of participants’ daily lives so that the case can be understood in-depth, so its complexity and context can be grasped [78]. As per the recommendations of Yin [79], multiple sources of evidence were used for this case study, including individual and group interviews, non-participant observations, and a thorough analysis of organizational documentation. Several data collection techniques were used to triangulate the data and ensure the validity of the research as well as minimize bias in the collected data [78].

Data was collected at the workplace of the people who had volunteered to participate in the study, which included four psychosocial workers, six top managers (directors), and five middle managers. Each interview lasted 60 min and was based on a semi-structured interview guide that covered topics such as their reflections on leadership practices during the deployment of the IPMS in the context of service integration. Group interviews were also conducted and lasted 90 min. Group interviews provided important validation of the interpretation of the data collected during the individual interviews [80]. Participation in the focus groups was not limited to people who participated in the individual interviews and was open to members of their teams. Over 350 h of non-participant observations were collected during different meetings with managers and staff, and organizational documentation was also consulted.

Based on the different data sources, the analysis strategies used gave rise to a detailed description of the case. These strategies included three major coding steps, i.e. open coding, axial coding, and selective coding [81, 82]. Using the NVivo software, open coding allowed meaning units to be grouped based on the theoretical context of the research. The flexibility and openness of the researchers also allowed new categories to emerge depending on what the participants had to say. Axial coding was then carried out, which allowed for an in-depth conceptual analysis of the relationships between the different categories identified on the open coding grid. The final step in the analysis was selective coding. Some categories were grouped into a tree map that reflected the structure of ideas and relationships and that revealed key themes [82].

### Results and practical contributions

#### Setting the stage

In June 2017, after the institutional merger, the executives and top managers of the institution launched a large-scale rapid improvement event to promote better coordination of services between the youth programs directorate and the youth protection directorate.^3^ This first meeting, initiated by the organization’s strategic team, was seen as an opportunity to replace existing practices with new ones. The executives’ (CEO and COO) invited four directors and four middle managers to two consecutive days of reflection on how to improve the accessibility and continuity of services for children in a given territory. The CEO took on transformational leadership and acted in line with the organization’s orientations and the new reform whose main goal was to integrate services.

On the first day of the large-scale rapid improvement event, the middle managers were asked to map the current service trajectory of a child and the child’s family. This collective exercise identified areas of criticality and let staff reflect on the causes of the problem. A root-cause analysis revealed over 60 causes in six categories, which included roles and responsibilities, service organization, work organization, competencies, management tools, and communication tools. At the end of the first day, some participants (middle managers) felt uncomfortable, while others were very much in denial about the service gaps observed based on the child’s pathway through services over 10 years.

On the morning of the second day, after listening to middle managers about their unease, the CEO set out a vision that expressed her expectations for services provided to youth clients. This vision was based on major strategic orientations and collaboration as a chosen organizational value. As one director pointed out:

“I don’t think the project would have worked without the CEO’s clear vision.”

The CEO exhibited transformational leadership by articulating a clear vision for the service integration project. The commitment of the executives was a critical success factor in the implementation of an improvement methodology [83], as the CEO positively communicated her vision while reinforcing her managers’ motivation and confidence. The vision of the integration project was as follows:

- Young people will never suffer alone again.
- Young people will always receive the appropriate support in their pathway.
- Do the impossible to avoid putting the development or safety of a child in danger.^4^

This vision played a decisive role not only in the implementation of the service integration project but also throughout the project itself. Transformational leadership encourages managers to seek out extraordinary goals [84] in terms of accessibility and continuity of services. The large-scale rapid improvement event coupled with the CEO’s transformational leadership encouraged the implementation of integrated teams to support service integration solutions.

To reinforce collaboration between directors and middle managers, a strategic A3^5^ report titled “Jimmy” was developed after the end of the large-scale rapid improvement event, and each middle manager at the meeting signed this report as a formal commitment to carry out and help implement the process. The Jimmy A3 was tracked every four weeks by the CEO in the strategic control room, during meetings of the senior management committee, to closely monitor the implementation of the action plan and measure the effectiveness of the key performance indicators. Based on the observations from the case study, the A3 report allowed for the decentralization of decision-making to start to emerge, which helped stakeholders get involved and participate in the integration project. A top manager at the meeting expressed thoughts about the commitment that everyone had to make:

“The A3 reports allowed people who were supposed to contribute to engage in reflection […;] when the managers signed the collaboration contract at the beginning of the project […,] that’s when people really became engaged.”

From the strategic A3 report, four other A3 projects were developed using rapid improvement events and were named Charlotte, Henriette, Rosalie, and Juliette. These activities required more tactical and operational approaches, and the goal was to develop an action plan to help integrate services in the desired trajectory. To monitor these A3s, operational control rooms were quickly deployed in the integrated youth teams, where employees, directors, and middle managers were brought together every week to set targets for the service integration project.

The operational control rooms of the new integrated youth teams provided part of the foundation for the emergence of distributed leadership, by democratizing management activities, i.e. team members take turns leading the activity, and this task is no longer under the purview of the manager. As one middle manager explained about the team’s ownership and accountability:

“During the summer […,] I had a team member take over to get us to the point that the room was part of our team […] and all employees had taken ownership of it.”

This reality was further unpacked by the following statement from a staff member:

“Since we became an integrated team, it has been much easier for us to work together and in proximity to each other. It’s been really positive.”

From the start of activities in the operational control rooms, the participants submitted many improvement initiatives [85], which demonstrated their willingness to help develop solutions that could improve the organization’s work and performance. For example, one staff member was dissatisfied with the wait times between assessments and the start of case management. The staff member submitted an improvement initiative to the operational control room so that the team could find solutions to reduce wait times and improve access to services for Jimmy. As the team manager pointed out:

“We dramatically reduced the wait time between assessment and the start of case management.”

Initial staff engagement in the integration project appeared good, especially following efforts made to urgently remove daily irritants that had persisted for many years. However, once these irritants had been dealt with, improvement initiatives became increasingly rare or addressed routine issues, such as poorly soundproofed consultation rooms. One middle manager said, “These are minor annoyances.” After one year, no improvements had been made on the key performance indicators, which indicated that problem-solving to find solutions to trajectory-related challenges was unfortunately not taking place.

#### Laissez-faire leadership: an obstacle for Jimmy

The project encountered major difficulties during the first year not so much due to the project managers’ commitment and belief in the project’s rationale but because of how performance indicators were monitored. According to one middle manager, performance measurement was one of the main reasons for low levels of engagement from staff, who felt that quantitative evaluation did not properly represent the fundamentally human dimension of their jobs:

“I think there are challenges in making the numbers reflect the living reality of the service offer.”

Observations from the research showed a laissez-faire leadership style on the part of some directors who experienced difficulties engaging their teams in measuring indicators. Comments from one staff member speak to this problem:

“Take away the statistics, performance, and everything. They are completely useless. It just puts pressure on staff.”

The action plans for the A3 projects, therefore, remained conceptual and were not conducive to quick action. An informal status quo bogged down the project in a kind of non-performance, and the service trajectory was not monitored at all. At this point, it was observed that some leaders with formal power over the team were negatively influencing the project.

#### Transactional leadership to the Rescue of the Integration Project

After observing the directors’ laissez-faire style, the CEO and her associate (COO) organized a project review day in June of the following year. They started the meeting by sharing their disappointments and communicating their reservations about any real progress having been made with the service integration project with the various middle managers involved since the project started in June 2017. The integration project was not bearing the expected fruits. The directors and middle managers found it difficult to be confronted and questioned about why collaborative spaces were being hindered. However, as one middle manager expressed:

“The meeting was key for the project. It gave a glimmer of hope [for us to] work more as a team. We had to look at what wasn’t working.”

Aware that the philosophy of the IPMS reinforces a bottom-up approach to problem-solving to help staff identify problems and find the right solutions, the CEO set up a status sheet so that everyone could become better aware of the trajectory and could more effectively document it. Every week, using the status sheet, a performance review was conducted by monitoring the effectiveness of the interfaces of the trajectory through accessibility and continuity indicators. This action by the CEO embodied a more directive transactional leadership approach aimed at getting the project back on track. At first, middle managers were reluctant to adopt this practice with their teams because they felt obligated to apply top-down decisions. However, it proved to be a turning point toward the success of the integration project. As one middle manager explained:

“I feel like the status sheet is what changed things.”

The inevitable centralization of leadership toward the executives led to a genuine renewal in the deployment of the IPMS so that the trajectory targets could be achieved. Furthermore, the difficulties encountered during the first year of the integration project and the subsequent changes implemented by the CEO contributed to the departure of two key opinion leaders (directors) who did not agree with the organizational priorities expressed by the executives.

Status sheet sessions were held weekly during a Friday morning conference call attended by all middle managers. The observations suggest that transactional leadership was an excellent vehicle for leading the status sheet, as this tool is task and results-oriented. The sessions began with a review of indicators related to accessibility, continuity, and quality so that everyone could quickly detect any deviations from the standard. Leaders could focus on generating possible solutions from an employee in order to have corrective action taken. However, the leader (director) could also help collaboration emerge during the second part of the status sheet, which focused on qualitative aspects, particularly the perceptions of the team climate, risk management, and comments received by users.

Without calling it a recognition program as such, team members’ positive performance was highlighted by their peers every week both during the control room activities and during the status sheet sessions. The purpose of this recognition was to improve the working climate so that each team member felt valued, which was appreciated by both middle managers and employees:

“The other thing I really like is how good performance is recognized. This is very positive because we never used to take the time to do this every week. It happened maybe once or twice a year. We definitely point it out and we have peers recognize each other. Peer recognition increases team spirit.”

After six months of using the status sheet in conjunction with the control rooms, significant improvements to accessibility and continuity had been achieved. Initially, the average wait time in child protection was 16.96 days, compared to the government target of 12 days. The achieved result was 8.35 days. Service intensity at the start of the project was 0.96 interventions per day per worker and improved to 3.6 interventions per day per worker. Significant gains were also achieved regarding the implementation of intervention plans, which rose from 70% to the government target of 85% and from 10 to 43% in statutory and voluntary contexts respectively. Furthermore, the disability insurance rate decreased to 8.70% from its initial level of 14.30%. The target was 6.03% at the time.

#### New leaders, new dynamic

As mentioned previously, two directors left and were replaced during the first year of the integration project. This change in top management for the youth programs led to a new dynamic that supported greater interprofessional collaboration, particularly in the control room. After this change, leadership started to be distributed based on trust and based on the managers’ mutual knowledge of each other. As one middle manager pointed out:

“We’ve got a director of youth protection and a director of youth programs working side by side, and that’s a big win.”

When the new directors arrived, they began sharing leadership and moved toward better collaboration that left no room for interdepartmental competition, which confirms that service integration is more dependent on human factors and added value for the client experience rather than on organizational and structural issues [86]. The two directors distributed the same values and same goals for improving youth services offered in the territory. Both directors were aware of the need to collaborate in an exemplary way, and they decided to create a single tactical control room to create a fluid trajectory of care and services for the territory’s children, youth, and families. In the interviews, one of the new directors said:

“You have to be completely in sync. Since the fall, we haven’t felt that the two departments were working as well together, [nor did we have anything] facilitating a strong feeling of cohesion in our control room.”

The two new directors supported the clear and integrated vision of Jimmy’s trajectory articulated by the CEO and upheld the need for measurement to achieve the performance targets. Meetings in the tactical control room provided opportunities for both departments together to evaluate team performance by sharing essential information to raise important issues about Jimmy’s trajectory and devise collective solutions through consensus, thus supporting Barnas’ view [75] that the control room can become a vehicle for knowledge sharing, team building, and continuous improvement.

Given that no formal activities were held in the tactical control room during the first year of the project’s deployment, the new directors had to take ownership of how the tactical control room functioned. The decision that the two departments with the same clientele should share the same control room led to a change in leadership and gave the teams the legitimacy to commit to the service integration project. The goals that the directors shared with the teams to help everyone understand the reasoning behind the changes created a climate of mutual trust, which was a key factor that allowed distributed leadership to emerge after two years of experimentation. Democratizing control room activities also fostered the emergence of distributed leadership. Both directors made this a collective responsibility. Other departments were also involved in Project Jimmy. Managers or staff were invited to attend control room activities to find collective solutions to the problems of accessibility, continuity, and quality throughout Jimmy’s trajectory. Distributed leadership made it possible to develop a culture of integration between the first and second service lines. For example, as a director pointed out:

“Implementing the tactical room jointly between the youth program and the youth protection directorate has advantages, such as continuity [...;] there are projects that, even though they are more the responsibility of the other department, considering that I am involved, I take responsibility for them.”

## Discussion

**Fig. 1:**
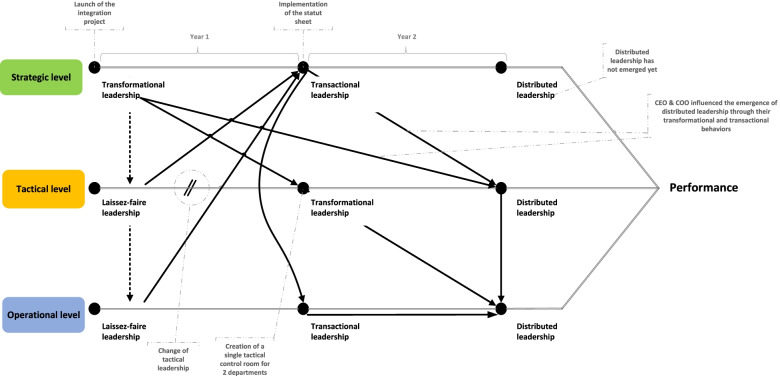
Evolution of leadership styles

Distributed leadership is considered an effective way to foster team performance in complex situations, such as those found in programs for children and youth in difficulty. This paper tracks the evolution of the different leadership styles adopted during the implementation of a new management model (Fig. 1). The results show the importance of transformational and transactional leadership as contributors to the emergence of distributed leadership in this type of project. They also highlight how good leadership can create the basis of better coordination among team members [59].

At the onset of the integration project, the CEO exhibited transformational leadership by articulating a clear vision, by challenging directors and managers to actively participate in the integration project, and by fostering their participation by signing the Jimmy A3. Transformational leadership exhibited by top administrators is frequently cited as behavior that allows institutions to evolve through continuous improvement [87]. The transformational leadership adopted by the executives nevertheless had little impact on the development of distributed leadership on a strategic level. Our observations also suggest that the post-merger environment and fiscal austerity at the time hindered the emergence of a new leadership model. For example, the remaining middle managers were destabilized in the face of their new, larger, and more complex responsibilities. They felt lost inside a multi-site managerial structure and clinical management of two irreconcilable professional fields. This resulted in passiveness, waiting for clear directions from top management on how to deploy with their new teams.

The matrix organizational structure implemented because of the new reform seemed to have negatively impacted the development of collaboration, as the leader with formal power had a purview that was too broad. This partially explains why distributed leadership at the strategic level still had not fully emerged by the end of our study.

This case study demonstrates that transformational leadership from executives did not initially influence the type of leadership adopted at the tactical level, as highlighted by the dotted arrows in Fig. 1. This was in part due to a lack of engagement from directors and middle managers that resulted in low task complexity and a lack of urgency in improving performance. This observation supports Kerr and Jermier’s argument [88] that such issues may hinder the emergence of distributed leadership. Our observations show that it took over a year for transformational leadership to be adopted by new leaders, by using more contemporary management practices focused on teamwork, service integration, and the sharing of the vision statement. The departure of top managers with a laissez-faire leadership style gave new life to the project.

Laissez-faire leadership led to the emergence of transactional leadership from the CEO, through the implementation of the status sheet, as a response to address the absence of progress made in the project. This course of action is typically more conducive in crises [89]. Transactional leadership played an operative role to help the team pursue activities related to the trajectory and to set previously non-existent measures. Low team maturity at the strategic and tactical levels, caused by laissez-faire leadership by some top managers, slowed down service integration processes. The arrival of two new directors with a strong desire to work together allowed distributed leadership to emerge more quickly. This organizational context, therefore, created the necessary conditions to deploy the single tactical control room and monitor Jimmy’s trajectory. Our findings stress the importance of member familiarity and maturity in reducing the time it takes for a team to get its bearings, establish its work standards, and accelerate its development [90, 91]. The development of distributed leadership is also favoured by a tactical team composed of managers possessing interdependent clinical knowledge [92]. Hence, the implementation of a unique control room where influence and leadership roles were shared allowed for collaboration to form quickly at the operational and tactical levels.

The executives used the reform as an opportunity to create territorial teams dedicated to children, youth, and families. The pooling of professional and multidisciplinary expertise and the initial familiarity of members contributed to the emergence of distributed leadership. From the start of the project and the set-up of the control rooms, each member’s contribution to providing accessible services was an important mechanism for achieving the required interdependence to develop distributed leadership [93]. Our observations show that familiarity between team members greatly reduces the time required to adopt a collaborative posture, which is in keeping with the research of Adams et al. [90].

Despite good proximity, a certain degree of familiarity, and high task complexity, our findings show that the teams still seemed to wait for a leader to provide solutions to their problems. The control room lets staff identify problems and propose appropriate solutions. The control rooms, particularly the operational control rooms, therefore became more like information rooms. According to Pearce and Sims [91], the basic prerequisite to allow distributed leadership to develop is a willingness of team members to become actively involved and to want to share responsibility. The respondents’ observations and comments did not indicate a desire to become actively involved in the team to the extent that they wanted to participate in leadership functions. However, they did appreciate receiving organizational information every week from the control rooms. The middle managers tried their best to adopt new management behaviors through the control room activities and status sheet sessions by adopting a management style that reconciles transformational and transactional leadership styles with a new style based on distributed leadership.

The implementation of an IPMS can support a new way of managing organizations where leadership and decision-making are shared in teams at all levels of the organization. Notwithstanding this willingness to share and adopt distributed leadership through collaborative practices, a more traditional top-down management style is a fundamental source of decision-making for organizations to run smoothly.

In the end, some leadership responsibilities have been retained by the department head while other responsibilities are now distributed among the integrated team through the control rooms and status sheet. Distributed leadership is more in keeping with collaborative profiles or competencies that are strongly ingrained in individuals. The complementarity between transformational leadership and distributed leadership at all hierarchical levels is a promising avenue to reinforce the organization’s capacity to change. The more that this leadership combination evolves, the more the organizational capacity to change will improve as new top and middle managers with a collaborative style arrive and real improvements are made in terms of care and service accessibility, and continuity.

Overall, this case study shows that a service integration project does not need to be guided by a single leadership style but rather by distributed leadership that emerges over time. This study also highlights the interdependence of leadership styles, where one is usually the result of another. While distributed leadership did not emerge quickly, its foundations were laid at the onset of the integration project. The CEO recognized the complexity of the project to integrate psychosocial services and that she needed to work with people who had skills and expertise that were complementary to her own (*recognizing individuals*). Through transformational leadership, she asked top and middle managers to contribute to this project, based on their knowledge and expertise in the field of services for children. This would ultimately legitimize the emergence of distributed leadership further down the line, even though the short-term effects were not conclusive.

How long it takes for this leadership to emerge will depend on not only the organizational context but also the desired level of interdependence. The literature on the emergence of different types of leadership does not mention a minimum timeframe required to establish collaboration between the members of a work team through the use of the different tools of the IPMS. This study was conducted in a chaotic context due to the merger of institutions, the reduced number of managers, and the resulting insecurity among staff. However, this study reveals that the emergence of distributed leadership must be considered from a multidimensional standpoint. Tools are not the only aspects that will create optimal collaborative conditions, as issues of power and influence can also seriously hinder integration projects in which the desired level of interdependence is contingent on leadership behaviors.

## Conclusion

This research demonstrates the importance of the contribution of various leadership styles (transformational, transactional, and distributed) in the deployment of an IPMS. This study was carried out in the child, youth, and family social services sector, where different hierarchical levels, i.e. both managers and staff, were required to adopt new behaviors so that distributed leadership could emerge. This qualitative study makes a useful contribution regarding the complementarity of the foundations of distributed leadership, the tools of the IPMS, and integration processes applied over time.

This case study raises questions about its transferability, along with certain limitations. The scope of this study was limited to the youth sector. A comparison with other sectors could be useful. Future research could be done in similar contexts (such as other similar healthcare institutions) to explore the conditions and barriers for success, and for organizations to learn collective lessons to improve the performance of health and social services networks. Our results also show that recognition of team performance may have helped trigger the emergence of shared leadership. Hence, future research could explore this phenomenon through a positive psychological lens [94], to study how recognition of performance influences the emergence of shared leadership. Future studies could also explore the emergence of distributed leadership through the lens of change management, and from the standpoint of performance improvement. The IPMS could represent a condition in which distributed leadership works and positively affects team performance. The required attitudes and skills that staff need to sustain the integrated performance management system over the long term and primarily support the sharing of leadership among all team members could also be explored.

## Data Availability

The datasets generated and/or analysed during the current study are not publicly available due to protect the confidentiality of respondents and to comply with ethical commitments. Aggregated data is available from the corresponding author on reasonable request.

## Footnotes

1 MSSS strategic plan (Goal 19): Improve governance and reduce the network’s management structure by reducing 1300 full-time equivalent management positions by 2017-2018.

2 MSSS strategic plan (Goal 20): Promote management that focuses on performance improvement. By 2018, all institutions will have deployed their strategic control rooms.

3 The youth programs directorate provides first-line services (health and social services) to children and youth aged 0 to 18 years and manages inpatient and outpatient rehabilitation programs and the application of measures (section 33) delegated by the youth protection directorate. The mandate of the youth protection directorate is to protect children and adolescents whose security or development is compromised. It responds to reports received from individuals who have reason to believe that the development or safety of a young person aged 0 to 17 is in jeopardy.

4 Children must receive the full range of services to ensure their development and safety.

5 The A3 report is a summary document that conveys information about projects on a single A3-sized sheet (11X17 format in North America) on which a problem-solving approach is expressed in a structured way.
